# Structural and Photoelectronic Properties of κ-Ga_2_O_3_ Thin Films Grown on Polycrystalline Diamond Substrates

**DOI:** 10.3390/ma17020519

**Published:** 2024-01-22

**Authors:** Marco Girolami, Matteo Bosi, Sara Pettinato, Claudio Ferrari, Riccardo Lolli, Luca Seravalli, Valerio Serpente, Matteo Mastellone, Daniele M. Trucchi, Roberto Fornari

**Affiliations:** 1 Istituto di Struttura della Materia, Consiglio Nazionale delle Ricerche (ISM–CNR), Sede Secondaria di Montelibretti, DiaTHEMA Lab, Strada Provinciale 35D, 9, 00010 Roma, Italy; sara.pettinato@unicusano.it (S.P.); valerio.serpente@ism.cnr.it (V.S.); daniele.trucchi@ism.cnr.it (D.M.T.); 2 Istituto dei Materiali per l’Elettronica e il Magnetismo, Consiglio Nazionale delle Ricerche (IMEM–CNR), Parco Area delle Scienze 37/A, 43124 Parma, Italy; matteo.bosi@imem.cnr.it (M.B.); claudio.ferrari@imem.cnr.it (C.F.); rcrd.lolli@gmail.com (R.L.); luca.seravalli@imem.cnr.it (L.S.); roberto.fornari1@unipr.it (R.F.); 3 Faculty of Engineering, Università degli Studi Niccolò Cusano, Via Don Carlo Gnocchi 3, 00166 Roma, Italy; 4 Department of Physics and Earth Science, Università di Ferrara, Via Saragat 1, 44122 Ferrara, Italy; 5 Department of Mathematical, Physical and Computer Sciences, Università di Parma, Parco Area delle Scienze 7/A, 43124 Parma, Italy

**Keywords:** wide-bandgap semiconductors, gallium oxide, diamond, vapor phase epitaxy, optical microscopy, X-ray diffraction, electrical characterization, spectral photoconductivity

## Abstract

Orthorhombic κ-Ga_2_O_3_ thin films were grown for the first time on polycrystalline diamond free-standing substrates by metal-organic vapor phase epitaxy at a temperature of 650 °C. Structural, morphological, electrical, and photoelectronic properties of the obtained heterostructures were evaluated by optical microscopy, X-ray diffraction, current-voltage measurements, and spectral photoconductivity, respectively. Results show that a very slow cooling, performed at low pressure (100 mbar) under a controlled He flow soon after the growth process, is mandatory to improve the quality of the κ-Ga_2_O_3_ epitaxial thin film, ensuring a good adhesion to the diamond substrate, an optimal morphology, and a lower density of electrically active defects. This paves the way for the future development of novel hybrid architectures for UV and ionizing radiation detection, exploiting the unique features of gallium oxide and diamond as wide-bandgap semiconductors.

## 1. Introduction

Wide bandgap semiconducting (WBS) materials, namely semiconductors with an energy bandgap *E_g_* > 2 eV, offer significant advantages over conventional ones (e.g., Si, GaAs), especially for applications in which a higher operating temperature and voltage are required. WBS are therefore ideal active materials for high-power high-frequency electronics [1,2], detection of ionizing radiation in harsh environments [3], high-speed data transfer and storage [4], i.e., all applications where devices based on conventional semiconductors do not ensure optimal performances and robustness.

Among WBS, gallium oxide (Ga_2_O_3_) and diamond stand out for their ultra-wide bandgap, being in the range 4.6–5.2 eV for Ga_2_O_3_ (depending on the considered polymorph) and equal to 5.5 eV in the case of diamond. Similar values of energy bandgap imply similar optical absorption properties, as well as close energy values for electron-hole pair creation under ionizing radiation (approximately equal to *E_i_* = 2*E_g_* + 1.43 according to an empirical model [5] used to successfully predict *E_i_* for most semiconductors). For this reason, high-performance optoelectronic devices where solar blindness and/or extremely low dark current are mandatory requirements, such as high-responsivity deep-UV photodetectors for space and safety applications [6,7,8] or fast-sensitive detectors for X-ray imaging and dosimetry [9,10,11], have been equivalently developed with both Ga_2_O_3_ and diamond as active materials. On the other hand, some properties of Ga_2_O_3_ and diamond can be considered complementary in certain respects. For instance, two of the major drawbacks of Ga_2_O_3_ are the poor thermal conductivity (10–30 W m^−1^ K^−1^) [12] and the unfeasibility of p-type doping; conversely, diamond has the highest thermal conductivity among solid materials (about 2200 W m^−1^ K^−1^) [13] and p-type conductivity is easily achievable, but an efficient n-type doping is still far from being obtained [14]. Moreover, diamond has a unique feature among semiconductors, i.e., tissue equivalence [15], making it the ideal material for radiation therapy dosimeters [10,11]. This has raised interest in the development of Ga_2_O_3_/diamond heterostructures in the past few years, especially in the field of high-power density electronics, where there is a strong need for complementary logic devices able to avoid excessive heating, which would be detrimental to the device’s performance, reliability, and lifetime [15].

Several studies have been reported in the literature on the growth of Ga_2_O_3_ films on diamond substrates, employing different techniques such as metal–organic chemical vapor deposition (MOCVD) [16], atomic layer deposition (ALD) [17], radiofrequency magnetron sputtering [18], and low-pressure chemical vapor deposition (LPCVD) [19]. Microwave-plasma-assisted chemical vapor deposition (MWCVD) of polycrystalline diamond films on free-standing Ga_2_O_3_ substrates has also been reported [20,21], further demonstrating the growing interest in hybrid Ga_2_O_3_/diamond devices and their potentialities. The above-mentioned works are specifically focused on monoclinic β-Ga_2_O_3_, which, among the five polymorphs of Ga_2_O_3_ (α, β, γ, δ, and κ), is the most thermodynamically stable at standard temperature and pressure. However, the other polymorphs have recently demonstrated that they deserve attention, as they even outperform β-Ga_2_O_3_ in some specific applications. For instance, the corundum α-phase shows a wider bandgap (5.2 eV), making it more attractive than β-Ga_2_O_3_ for power electronics [22], whereas the orthorhombic κ-phase allows obtaining solar-blind UV-C photodetectors with superior performance [23], as well as X-ray detectors with a higher sensitivity and stability over time than their β-Ga_2_O_3_-based counterparts [24].

The growth on diamond substrates of Ga_2_O_3_ films with a crystalline phase different than the monoclinic (β) one has not been reported yet. Indeed, heterostructures based on α- or κ-Ga_2_O_3_ films have been mostly developed starting from *c*-plane sapphire (Al_2_O_3_) substrates, ensuring a good trade-off between cost effectiveness and lattice mismatch [25]. In this work, we explore for the first time the possibility of growing orthorhombic κ-Ga_2_O_3_ films on polycrystalline diamond substrates. We will show that metal-organic vapor phase epitaxy, followed by slow cooling under a controlled He flow, is the key process to obtain high-quality polycrystalline κ-Ga_2_O_3_ films with improved adhesion to the diamond substrate, low electrical resistivity, and good photoelectronic and charge transport properties. Therefore, the results of this work mark the first step towards the future development of novel hybrid κ-Ga_2_O_3_/diamond architectures for UV and ionizing radiation detection, in which the excellent sensitivity of κ-Ga_2_O_3_ can act synergistically with diamond high thermal conductivity for operation in harsh environments or with diamond tissue equivalence for real-time X-ray dosimetry with no need for external calibration.

## 2. Materials and Methods

Two commercially available polycrystalline “thermal management grade TM180” CVD diamond substrates (Element Six, Didcot, UK), with lateral dimensions of 10 × 10 mm^2^ and 300 μm thickness, were selected for the present work. Before the thin film deposition process, the diamond substrates were subjected to the following cleaning procedure: (1) acid cleaning in a HClO_4_:H_2_SO_4_:HNO_3_ mixture (concentration ratio 1:1:1) at boiling point for 20 min to remove possible non-diamond phases; (2) acid cleaning in *aqua regia* (HCl:HNO_3_ mixture, concentration ratio 3:1) at boiling point for 5 min to remove metallic contaminants; (3) ultrasound sonication in hot acetone for 5 min to remove organic contaminants; (4) rinse in deionized water; (5) dry in pure N_2_ flow.

Metal-organic vapor phase epitaxy (MOVPE) of Ga_2_O_3_ thin films was performed on the two polycrystalline diamond substrates in a reactor with horizontal geometry, without substrate rotation, at 650 °C and at a pressure of 100 mbar. Trimethylgallium (TMG) and ultrapure H_2_O were used as precursors, using a H_2_O/TMG ratio of about 200 and a H_2_O flow-rate of 200 sccm. Helium (total flow-rate 400 sccm) was used as a carrier gas to deliver the precursors to the growth chamber. The deposition time was set to 15 min. The only difference between the two heteroepitaxial growth processes was in the post-deposition cooling step. The first sample was subjected to “natural” cooling (NC) by simply turning the furnace off, resulting in an average cooling rate of 15 °C/min in the first 5 min and a slower cooling down to room temperature. It is worth noting that the first minutes are the most significant from the point of view of thermal stress and point defect thermodynamics. A three-step very slow cooling (SC) procedure was followed for the second sample: (1) cooling from 650 °C to 500 °C in 2 h; (2) cooling from 500 °C to 300 °C in 1.5 h; and (3) “natural” cooling down to room temperature by turning off the furnace. In all the cases, cooling was performed under He flow and at a chamber pressure of 100 mbar.

Visual inspection through optical microscopy (ECLIPSE Ni-E equipped with a DS-Qi2 camera and 10×–100× objectives, Nikon, Tokyo, Japan) was performed to assess the film integrity after the deposition process, aimed at verifying the presence of cracks or other macroscopic defects.

X-ray diffraction (XRD) was employed to investigate the structural quality of the deposited films and the crystallographic phase of Ga_2_O_3_. Measurements were performed with a high-resolution X-ray diffractometer (X’Pert PRO, Philips, Amsterdam, The Netherlands) equipped with a Goebel monochromator and analyzer crystals on both incident and diffracted beams, able to reduce the chromatic dispersion to 10^−4^ and the angular resolution to 12 arcsec. The *K*_α_ fluorescence line (*K_α_* = 1.54 Å) of a Cu anode was selected as impinging radiation. High-angle measurements were collected in the 10° < 2*θ* < 120° angular range, and geometry was set to Omega-2 Theta scan mode.

In order to perform electrical and photoelectronic characterization, metal contacts were fabricated by radiofrequency (RF) sputtering deposition (LH Z400, Leybold GmbH, Köln, Germany) on the surface of the two Ga_2_O_3_ thin films. Before deposition, the samples were cleaned by ultrasound sonication in acetone and isopropanol for 5 min to remove possible organic contaminants introduced after XRD characterization, then rinsed in deionized water and dried in pure N_2_ flow. The sputtering chamber was evacuated down to a base pressure of 3.0 × 10^−6^ mbar, and then Ar gas was introduced up to a partial pressure of 2.5 × 10^−2^ mbar. Sputtering deposition was performed by using an Au target (purity 99.999%, Kurt J. Lesker, Jefferson Hills, PA, USA) and keeping the Ar plasma on for 5 min at a RF power of 130 W. The distance from target to substrate was 70 mm. The geometry of the metal contacts (two rectangular 4.2 × 1.6 mm^2^ pads separated by a 1 mm wide gap) was automatically defined by superimposing a 50 μm thick stainless steel shadow mask on the sample during deposition. The thickness of the Au contacts was measured to be 300 nm by means of an optical profilometer (ContourX-100, Bruker, Billerica, MA, USA).

**Figure 1:**
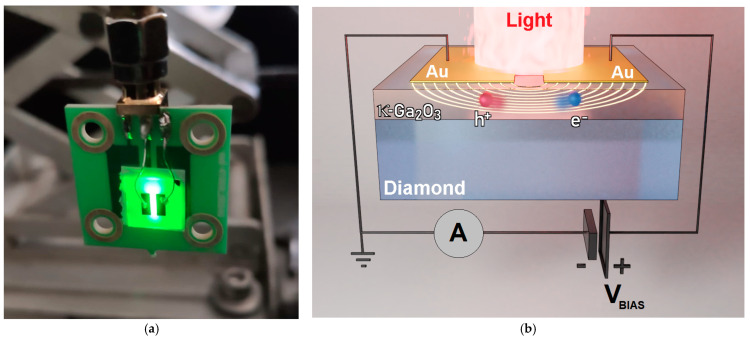
(**a**) Picture of the final device mounted on a PCB. Green light (546 nm wavelength) focused on the interelectrode gap was used for position adjustment before the spectral photoconductivity experiments; (**b**) Sketch of the operating principle of the device used for spectral photoconductivity experiments. The same device was also employed for current-voltage measurements (in dark conditions) to evaluate the electrical resistivity of the Ga_2_O_3_ film.

The metalized samples were then mounted on dedicated printed circuit boards (PCB), equipped with standard 50 Ω SMA (Sub-Miniature Version A) coaxial connectors. Subsequently, the Au contacts were wire-bonded to the two SMA terminal pads for biasing and signal collection. The complete on-board device was used for dark current-voltage measurements, performed by means of a precision electrometer (Model 487, Keithley Instruments, Cleveland, OH, USA) simultaneously acting as a voltage source and a current meter. Spectral UV-Vis-NIR photoconductivity experiments were performed in the range of wavelengths *λ* = 200–1000 nm by measuring the photocurrent generated by a monochromatic light beam focused on the 1 mm-wide interelectrode gap (Figure 1). Light was produced by a 200 W Hg(Xe) ozone-free lamp (Model 71229, Newport Oriel Instruments, Stratford, CT, USA) coupled to a dual-grating monochromator (Model Cornerstone 260, Newport Oriel Instruments, Stratford, CT, USA). To ensure better sensitivity to sub-bandgap wavelengths, measurements were performed in modulated-light mode using an optical chopper (Model SR540, Stanford Research Systems Inc., Sunnyvale, CA, USA) set to a frequency of 14 Hz. Modulated photocurrent signals were converted to voltage by a low-noise transimpedance amplifier (Model 181, Princeton Applied Research, Oak Ridge, TN, USA) and finally acquired by a lock-in amplifier (Model 5209, EG&G, Dallas, TX, USA). The bias voltage was applied to the devices by means of a Keithley 487 electrometer, exclusively used as a voltage source.

## 3. Results and Discussion

### 3.1. Optical Microscopy

**Figure 2:**
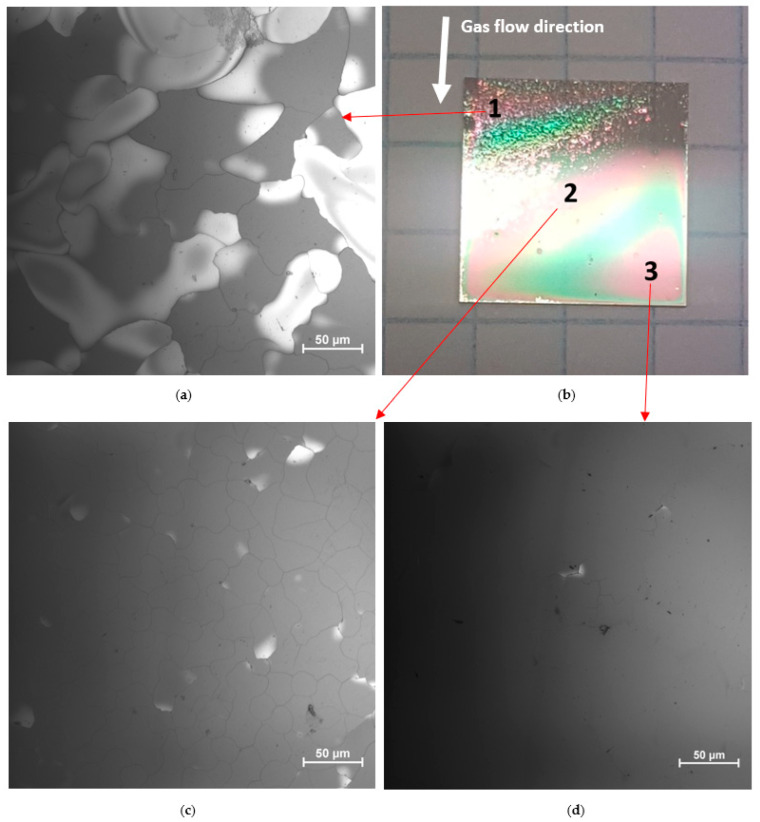
Optical microscopy images of the surface of the naturally-cooled sample: (**a**) details of the hazy zone (point 1); (**b**) overall view of the 10 × 10 mm^2^ surface; (**c**) details of the mixed zone (point 2); (**d**) details of the mirror-like zone (point 3).

Figure 2b shows the whole surface of the NC sample soon after the end of the cooling process. As can be seen with the naked eye, three different zones with different macroscopic aspects can be identified: a hazy zone (point 1), a mirror-like zone (point 3), and a mixed zone with small, defected areas within wide mirror-like areas (point 2). The presence of colored fringes indicates a thickness gradient of the Ga_2_O_3_ film; specifically, the thickness decreases from point 1 (740 nm) to point 3 (470 nm), whereas at point 2 it was measured to be 580 nm, corresponding to the average thickness of the film. The thickness gradient is due to the horizontal geometry of the MOVPE reactor, without substrate rotation; as a consequence, the deposition rate decreases slightly in the direction of the gas carrier flow (white arrow in Figure 2b). Therefore, the hazy zone is the thickest, because the upper edge faces directly the He flow, whereas the mirror-like zone is the thinnest. By looking more in detail using a larger magnification, the hazy zone (Figure 2a) is made up of an arrangement of cracks and wide grains (with sizes in the range 10–100 μm), whereas the mirror-like zone (Figure 2c) looks perfectly flat and homogeneous, with no cracks or other macroscopic defects. The mixed zone (Figure 2d) is substantially flat and homogeneous, with some micrometer-sized defects.

The overall impression is that the cracks observed on the surface in the hazy zone are due to some delamination, possibly driven also by the higher film thickness in this zone, as reported for other heterostructures [26,27]. Specifically, in the case of strongly bonded oxides [27], where dislocation formation and propagation steps are difficult, the thermal misfit stress gets more prominent. As a consequence, by increasing the thickness of the film, the strain in the film accumulates and causes cracking and delamination. For this reason, our hypothesis is that the huge mismatch between the thermal conductivity values of Ga_2_O_3_ [12] and diamond [13], implying very different coefficients of thermal expansion (1.5 × 10^−5^ K^−1^ and 1.0 × 10^−6^ K^−1^ at 300 K, respectively [28,29]), made the grown film peel off from the substrate during the cooling process. Indeed, when a very slow cooling was performed on the second sample to limit the detrimental effects of thermal mismatch, no signs of delamination were observed, even if the deposition time (and hence the expected thickness) was the same. Unlike the NC sample, the surface of the SC sample appears completely mirror-like to the naked eye (Figure S1b). Optical microscopy images taken from points 1 (Figure S1a), 2 (Figure S1c), and 3 (Figure S1d) all show a perfectly flat and homogeneous surface with no cracks or other macroscopic defects.

### 3.2. X-ray Diffraction

**Figure 3:**
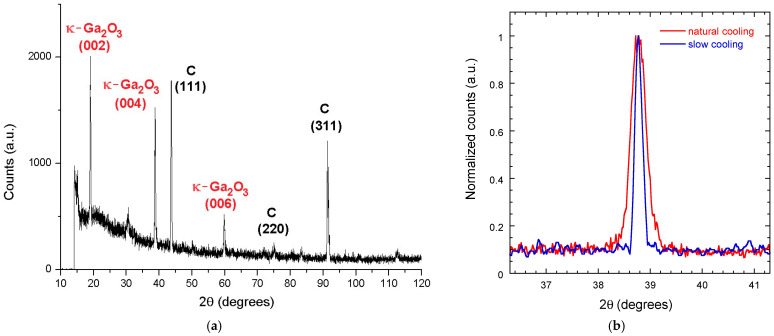
(**a**) XRD spectrum of the naturally-cooled NC sample; (**b**) Rocking curves of the 004 reflection for the NC sample (red curve) and the SC sample (blue curve). The mosaic spread, evaluated on the 004 reflection for both samples, is of the order of 1–5°.

The mirror-like zones were selected to perform XRD measurements on the two samples, the results of which are reported in Figure 3a for the NC sample. The κ-Ga_2_O_3_ reflections 002, 004, and 006, which are the fingerprints of the *c*-oriented orthorhombic phase of gallium oxide, are evident. The position of the peaks confirms previous XRD analysis of κ-Ga_2_O_3_ films grown by MOVPE on single-crystal (0001) sapphire substrates [30]. However, if compared to films grown on sapphire substrates, the peak intensity is about two orders of magnitude lower (e.g., 2000 cps vs. 100,000 cps in the case of the 002 reflection), clearly denoting a nearly polycrystalline nature of the κ-Ga_2_O_3_ epitaxial layer. This was actually expected considering the structurally poor quality of the polycrystalline diamond substrate, as confirmed by the relatively weak (the incident beam intensity is indeed of the order of 10^9^ cps) of the C(111), C(220), and C(311) diamond peaks, corresponding to the reflections of cubic diamond with an accuracy of ±0.05° [31].

A similar XRD spectrum (i.e., with the same diffraction pattern) was obtained for the SC sample, highlighting that the overall structural quality of the grown film in the mirror-like zones is not significantly affected by the cooling process, which is conversely crucial to ensure good film-substrate mechanical adhesion, to limit the occurrence of cracks, and to minimize interface defects. However, as shown in Figure 3b, reporting the rocking curves of the 004 peak, the FWHM (Full Width Half Maximum) evaluated for the NC sample (0.34°) is slightly higher than the value measured for the SC sample (0.16°), denoting the presence of a higher lattice strain.

### 3.3. Current-Voltage Measurements

**Figure 4:**
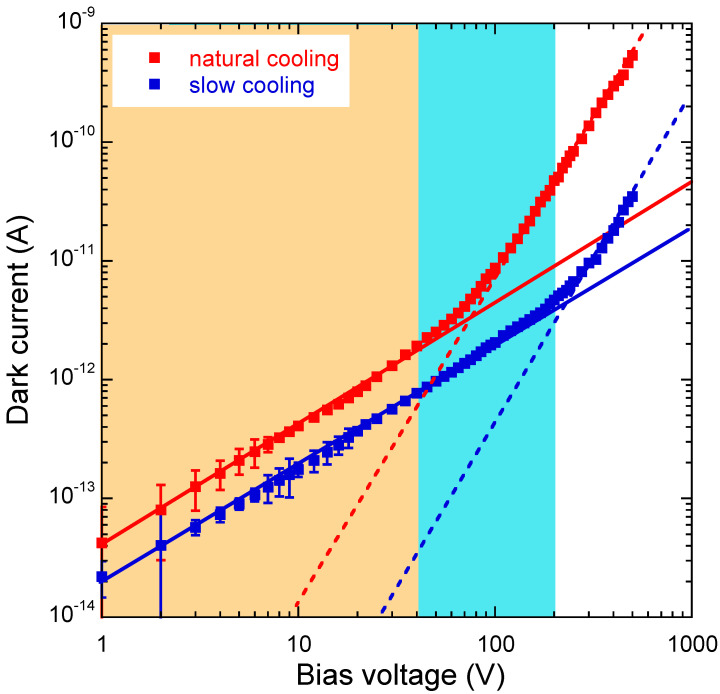
Log–log plot showing the current–voltage curves of the κ-Ga_2_O_3_ films measured under dark conditions. Orange and light-blue areas are a guide to the eye to visualize the range of ohmic charge transport for the NC and SC samples, respectively. Solid and dashed lines indicate the best linear and power-fitting curves for experimental data, respectively.

Figure 4 reports the current-voltage characteristics (log-log plot) measured for both samples in dark conditions in the range of bias voltages (*V_bias_* = 1–500 V). The first result to highlight is the higher dark current *(I_d_*) of the NC sample (red curve) than the SC sample (blue curve), denoting a higher density of electrically active defects, which are well-known to increase dark current in wide-bandgap semiconductors [32,33]. Among the possible defects responsible for the increased conductivity of the NC sample, we mention point defects possibly introduced by the faster cooling process and defects localized at the interface with the diamond substrate. Interface defects in Ga_2_O_3_ heterostructures have indeed been suggested to limit the performance of devices that require minimization of the dark current, such as photodetectors [34,35].

The ohmicity of Au contacts, as inferred from the best linear fit to experimental data (solid lines in Figure 4), is confirmed up to 40 V for the NC sample and up to 200 V for the SC sample, resulting in a dark resistivity value of *ρ_d_* = 6.01 × 10^9^ Ω cm and *ρ_d_* = 1.25 × 10^10^ Ω cm, respectively. Although both values are lower than that reported [24] on κ-Ga_2_O_3_ films grown on sapphire (*ρ_d_* = 3.15 × 10^10^ Ω cm), they nevertheless meet the threshold requirement for semiconductor-based X-ray detectors (10^8^–10^9^ Ω cm) [36].

At higher bias voltages (*V_bias_* > 40 V for the NC sample, *V_bias_* > 200 V for the SC sample), a non-linear behavior of the dark current is triggered. Specifically, in a similar way as reported on β-Ga_2_O_3_ [35] and other WBS [37,38,39], a trap-controlled space-charge limited current (TC-SCLC) regime is active in which a steep current increase is observed. The dark current-bias voltage relation follows a power law *I_d_* ∝ $V_{bias}^{m}$, where *m* is a coefficient related to the distribution of traps; specifically, *m* > 2 denotes the presence of an exponential band tail [39,40]. The log-log plot allows us to estimate the power coefficients *m* from the slopes of the best-fit curves (dashed lines in Figure 4), returning *m* = 2.53 ± 0.04 for the NC sample and *m* = 2.25 ± 0.10 for the SC one. Therefore, an exponential distribution of traps is present in both samples. However, the lower resistivity in the Ohmic regime and the lower bias voltage at which the TC-SCLC regime is triggered are both clear indications of a higher concentration of shallow traps induced when slow cooling is not performed after the film growth. Conversely, the density of deep traps (at least those contributing to charge transport) seems to be less affected by the cooling process, as can be inferred from the similar *m* values extracted at high *V_bias_* values for the two samples.

### 3.4. Spectral Photoconductivity Measurements

**Figure 5:**
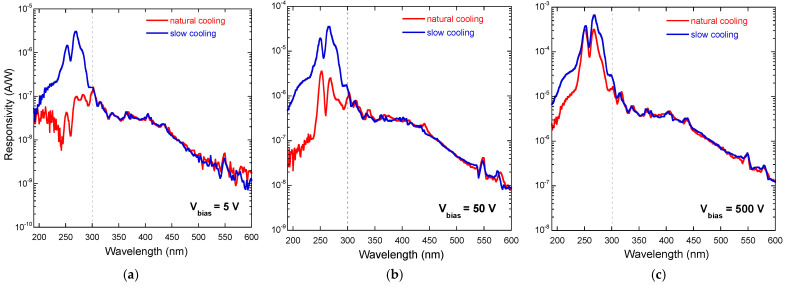
Spectral responsivity of the NC sample (red plots) and of the SC sample (blue plots) in the 200–600 nm wavelength range at different applied bias voltages: (**a**) 5 V, (**b**) 50 V, (**c**) 500 V. The dashed vertical lines indicate the wavelength (about 300 nm) separating the two identified photoconductivity regimes.

Spectral UV-Vis-NIR photoconductivity of the κ-Ga_2_O_3_/diamond heterostructures was measured in the range of bias voltages *V_bias_* = 1–500 V. Results are reported in Figure 5, showing the responsivity *R* of the two devices in three different cases: *V_bias_* = 5 V, 50 V, and 500 V. Responsivity was calculated as a function of the monochromator output wavelength *λ* as *R*(*λ*) = |*I_ph_*(*λ*)|/*P*(*λ*), where |*I_ph_*| is the modulus of the measured modulated photocurrent and *P* is the power of the radiation focused on the active area of the devices.

As can be seen from Figure 5, the responsivity curves of the two samples overlap almost perfectly for *λ* > 300 nm, independently of the applied bias voltage, whereas for *λ* < 300 nm, significant differences can be observed. Specifically, while the two signal peaks (the first one at 270 nm, corresponding to the bandgap of κ-Ga_2_O_3_, and the secondary one at 251 nm, presumably related to the presence of amorphous Ga_2_O_3_ within the epitaxial layer [41]) are always clearly distinguishable in the case of the SC sample, an apparent decrease in responsivity can be observed in the case of the NC sample, especially at low (5 V) bias voltage. At intermediate (50 V) bias voltage, the photoresponse for *λ* < 300 nm of the NC sample is partially recovered (the two peaks emerge clearly), but only when the applied bias voltage is high (500 V) is the amplitude of the signal peaks measured for the two samples comparable. 

**Figure 6:**
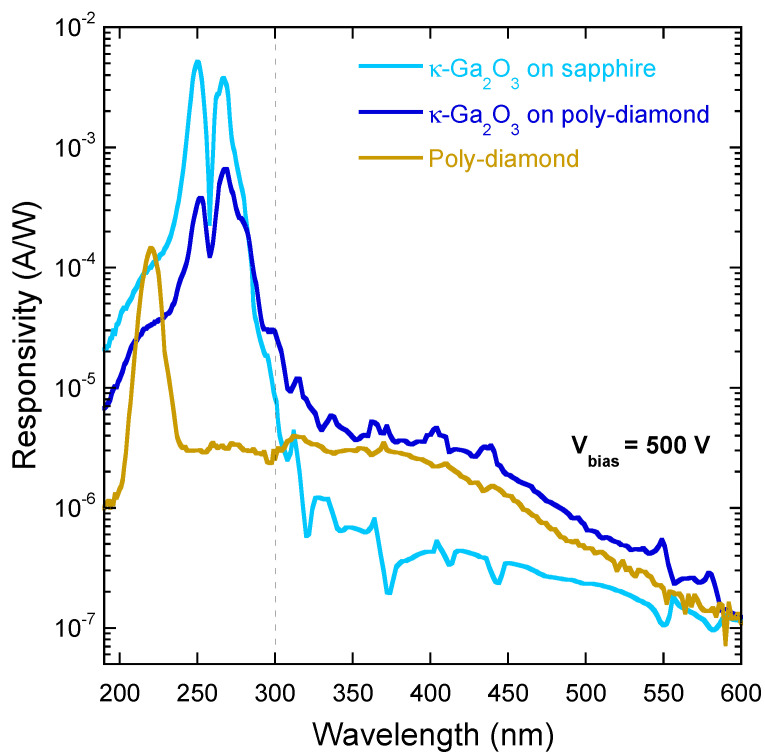
Spectral responsivity in the 200–600 nm range, measured at *V_bias_* = 500 V for: a κ-Ga_2_O_3_ epitaxial thin film (of approximately the same thickness as both NC and SC samples) grown on a high-quality single-crystal sapphire substrate (light blue curve), the κ-Ga_2_O_3_/diamond heterostructure (SC sample, blue curve), and a bare polycrystalline diamond sample of the same batch as those used as substrates for SC and NC samples (dark yellow curve). The peak at 225 nm in the poly-diamond curve is the signal peak related to the diamond bandgap. The dashed vertical line indicates the wavelength (about 300 nm) separating the two identified photoconductivity regimes.

In order to shed light on this behavior, it is useful to make a comparison (Figure 6) between the photoresponse of the κ-Ga_2_O_3_/diamond heterostructure and those measured in the same wavelength range (200–600 nm) for a bare polycrystalline diamond sample and for a κ-Ga_2_O_3_ thin film grown on a high-quality single-crystal sapphire substrate. Very significantly, we can observe that: (1) the curves measured for poly-diamond and for κ-Ga_2_O_3_-on-sapphire intersect at *λ* ≈ 300 nm; (2) the shape of the responsivity curve of κ-Ga_2_O_3_/diamond resembles that of poly-diamond for *λ* > 300 nm, whereas it retraces that of κ-Ga_2_O_3_-on-sapphire for *λ* < 300 nm. This can be understood by considering that light is completely absorbed by the κ-Ga_2_O_3_ thin film for *λ* < 300 nm, whereas for *λ* > 300 nm, the contribution of polycrystalline diamond absorption becomes dominant.

It is now possible to explain the differences observed in the responsivity curves of the NC and SC samples (Figure 5). When *λ* > 300 nm, photons are mostly absorbed within the polycrystalline diamond substrate, which is the same for the two samples, resulting in approximately the same responsivity. Conversely, when *λ* < 300 nm, photons are mostly absorbed within the κ-Ga_2_O_3_ thin film, so the lower responsivity measured for the NC sample is most probably due to a lower charge collection efficiency, caused by a significant trapping mechanism of the photogenerated carriers. When the applied bias voltage is high enough to trigger the de-trapping of carriers, the loss of responsivity is partially compensated.

**Figure 7:**
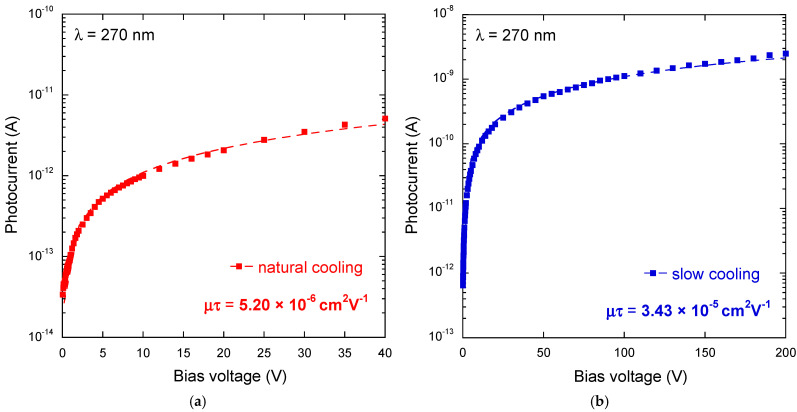
Amplitude of the modulated photocurrent signal as a function of the applied bias voltage for the NC sample (**a**) and the SC sample (**b**). Dashed lines show the best fit to experimental data obtained by using Equation (1). For a more accurate estimation of the *μ_e,h_ τ_e,h_* product with the Hecht’s model, which is strictly valid only in case of Ohmic behavior of the dark current, measurements were restricted to the 0–40 V range for the NC sample and to the 0–200 V range for the SC sample.

To further investigate the charge transport properties of the fabricated heterostructures, the modulated photocurrent amplitude at 270 nm was measured for both NC and SC samples as a function of the applied bias voltage in order to estimate the average mobility-lifetime product (*μ_e,h_ τ_e,h_*) of the photogenerated carriers according to Hecht’s equation [42]:

(1) $$I_{ph}\propto \frac{\mu_{e,h}\tau_{e,h}V_{bias}}{d^{2}}\left[1-exp\left(\frac{d^{2}}{\mu_{e,h}\tau_{e,h}V_{bias}}\right)\right]$$

where *d* = 0.5 mm is the average distance traveled by a photogenerated charge carrier (electron or hole) before being collected (i.e., half the spacing between the two Au electrodes on the top surface of the κ-Ga_2_O_3_ film). Results are reported in Figure 7. As can be seen, the mobility-lifetime product estimated for the NC sample (*μ_e,h_ τ_e,h_* = 5.20 × 10^−6^ cm^2^ V^−1^) is about one order of magnitude lower than the value estimated for the SC sample (*μ_e,h_ τ_e,h_* = 3.43 × 10^−5^ cm^2^ V^−1^). Being the mobility-lifetime product a powerful parameter to evaluate the charge transport properties of a semiconductor, it can be inferred that slow cooling is essential to maximize the charge collection efficiency of a κ-Ga_2_O_3_/diamond heterostructure. We have to observe here that the mobility-lifetime product found for the SC sample is, in turn, one order of magnitude lower than those reported on κ-Ga_2_O_3_ thin films deposited on single-crystal sapphire, all in the order of 10^−4^ cm^2^ V^−1^ [24]. However, this can be explained by considering the polycrystalline nature of the diamond substrates used in this work, which obviously poses a constraint on the crystalline quality of the deposited κ-Ga_2_O_3_ thin films with respect to the use of single-crystal substrates.

## 4. Conclusions

In conclusion, this work demonstrates that heteroepitaxial growth of orthorhombic κ-Ga_2_O_3_ thin films on polycrystalline diamond substrates can be successfully obtained by means of an optimized deposition process based on metal-organic vapor phase epitaxy at 650 °C, followed by slow cooling at reduced pressure under a constant He gas flow. Specifically, the slow cooling step turned out to be essential to preserving the integrity of the κ-Ga_2_O_3_/diamond interface by limiting the effects of the thermal mismatch, avoiding film peeling off, and preventing the formation of voids and other defects detrimental to the collection efficiency of the photogenerated charge carriers. It is worth mentioning here that a very low thickness of the κ-Ga_2_O_3_ film (e.g., 100 nm) may result in less delamination and cracks even after natural cooling, but it would also have a negative impact on the detection performance because of a lower photon absorption capability.

The obtained results represent the starting point for the development of hybrid κ-Ga_2_O_3_/diamond prototypes for deep-UV detectors for harsh environments and real-time direct-reading X-ray dosimeters. Directions for future research are based on a further refinement of both the deposition and the slow cooling process parameters, aimed at improving both the crystalline quality and the charge transport properties of the κ-Ga_2_O_3_ active layer. For this purpose, an extensive XRD topographic analysis, along with a parametric study of the photodetection performance as a function of the κ-Ga_2_O_3_ film thickness, will be performed. Moreover, the possibility of performing selective deposition for the definition of uncovered diamond areas to be used for calibration purposes under X-rays will be explored.

## Data Availability

The data that supports the findings of this study is available from the corresponding author upon reasonable request.

## Supplementary Materials

The following supporting information can be downloaded at: https://www.mdpi.com/article/10.3390/ma17020519/s1, Figure S1: Optical microscopy images of the surface of the slow-cooled sample: (a) details of zone 1; (b) overall view of the 10 × 10 mm^2^ surface; (c) details of zone 2; (d) details of zone 3. All the three zones have a mirror-like appearance.
