# Supplementary material for: Structural and Photoelectronic Properties of κ-Ga2O3 Thin Films Grown on Polycrystalline Diamond Substrates
Source: Materials (Basel). 2024 Jan 22;17(2):519. doi: 10.3390/ma17020519 (PMC10820879; doi:10.3390/ma17020519)
Supplement: Supplementary file 1 [file materials-17-00519-s001.zip › materials-2807625-supplementary.pdf]

Supplementary material

Structural and photoelectronic properties of κ-Ga_2O_3 thin films grown on polycrystalline diamond substrates

Marco Girolami, Matteo Bosi, Sara Pettinato, Claudio Ferrari, Riccardo Lolli, Luca Seravalli, Valerio Serpente, Matteo Mastellone, Daniele M. Trucchi and Roberto Fornari

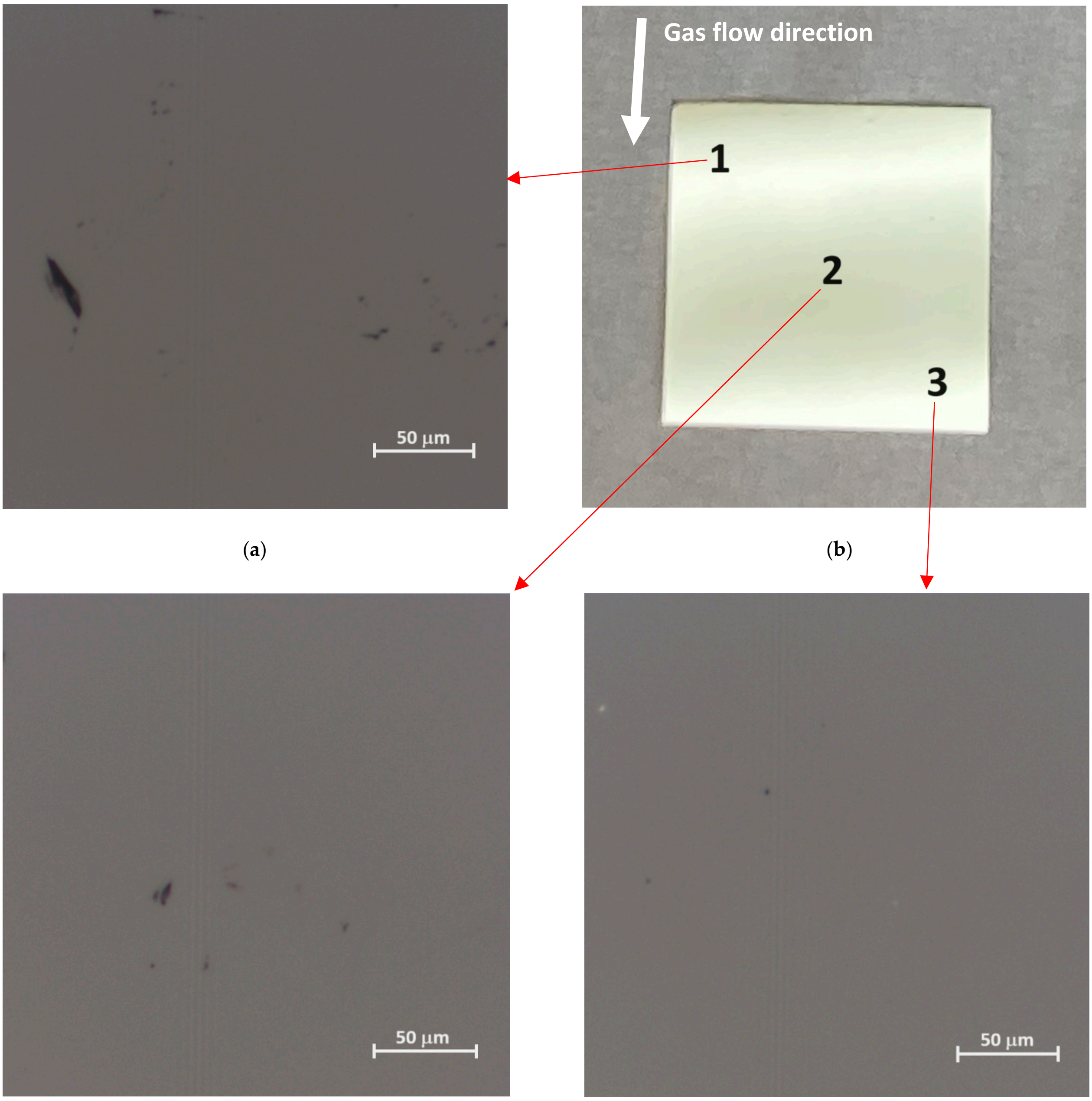

(**a**) (**b**)

(**c**) (**d**)

Figure S1. Optical microscopy images of the surface of the slow-cooled sample: (**a**) details of zone 1; (**b**) overall view of the 10 × 10 mm^2 surface; (**c**) details of zone 2; (**d**) details of zone 3. All the three zones have a mirror-like appearance.
